# Women as Sanitary Inspectors

**Published:** 1894-12

**Authors:** 


### Women as Sanitary Inspectors.

Dr. Moreau Morris, of the New York City Board of Health, is quoted in the New York *World* as having the utmost confidence in women of medical attainments as sanitary inspectors. Dr. Frances G. Deane has had two years' experience as a member of the sanitary corps, and Drs. Alice Mitchell and Helen Knight have recently been appointed to similar positions. Dr. Morris believes "that the fact of their being women will enable them to get into the homes of the poor where a man would be received with suspicion. The work that these women are engaged in is not the easiest, consisting as it does of a thorough canvass of the tenement houses in the district set apart for their work. They must visit every room, inspect sinks, peep into closets and climb innumerable stairs. Then, too, the people the sanitary physicians come in contact with are very apt to eye with displeasure the intrusion of strangers. On the whole, the work of the woman doctors of the Health Board is undeniably hard."

